# Machine learning-based prediction of microsatellite instability and high tumor mutation burden from contrast-enhanced computed tomography in endometrial cancers

**DOI:** 10.1038/s41598-020-72475-9

**Published:** 2020-10-20

**Authors:** Harini Veeraraghavan, Claire F. Friedman, Deborah F. DeLair, Josip Ninčević, Yuki Himoto, Silvio G. Bruni, Giovanni Cappello, Iva Petkovska, Stephanie Nougaret, Ines Nikolovski, Ahmet Zehir, Nadeem R. Abu-Rustum, Carol Aghajanian, Dmitriy Zamarin, Karen A. Cadoo, Luis A. Diaz, Mario M. Leitao, Vicky Makker, Robert A. Soslow, Jennifer J. Mueller, Britta Weigelt, Yulia Lakhman

**Affiliations:** 1 Departments of Medical Physics, Memorial Sloan Kettering Cancer Center, New York, NY USA; 2 Department of Medicine, Memorial Sloan Kettering Cancer Center, New York, NY USA; 3 Department of Pathology, Memorial Sloan Kettering Cancer Center, New York, NY USA; 4 Body Imaging Service, Department of Radiology, Memorial Sloan Kettering Cancer Center, New York, NY USA; 5 Gynecology Service, Department of Surgery, Memorial Sloan Kettering Cancer Center, New York, NY USA; 6 Department of Medicine, Weill Cornell Medical College, New York, NY USA; 7 Present Address: Department of Pathology, NYU Langone Medical Center, New York, NY USA; 8 Present Address: Department of Radiology, Sisters of Charity Hospital, Zagreb, Croatia; 9 Present Address: Department of Diagnostic Radiology, Japanese Red Cross Wakayama Medical Center, Wakayama, Japan; 10 Present Address: Department of Radiology, Trillium Health Partners, Mississauga, ON Canada; 11 Present Address: Department of Radiology, Candiolo Cancer Institute, FPO-IRCCS, Candiolo, Turin, Italy; 12 Present Address: Department of Radiology, Institute of Cancer Research of Montpellier (IRCM), INSERM U1194, Montpellier, France; 13 Present Address: Department of Radiology, Montpellier Cancer Institute, University of Montpellier, Montpellier, France

**Keywords:** Cancer imaging, Endometrial cancer

## Abstract

To evaluate whether radiomic features from contrast-enhanced computed tomography (CE-CT) can identify DNA mismatch repair deficient (MMR-D) and/or tumor mutational burden-high (TMB-H) endometrial cancers (ECs). Patients who underwent targeted massively parallel sequencing of primary ECs between 2014 and 2018 and preoperative CE-CT were included (n = 150). Molecular subtypes of EC were assigned using DNA polymerase epsilon (*POLE*) hotspot mutations and immunohistochemistry-based p53 and MMR protein expression. TMB was derived from sequencing, with > 15.5 mutations-per-megabase as a cut-point to define TMB-H tumors. After radiomic feature extraction and selection, radiomic features and clinical variables were processed with the recursive feature elimination random forest classifier. Classification models constructed using the training dataset (n = 105) were then validated on the holdout test dataset (n = 45). Integrated radiomic-clinical classification distinguished MMR-D from copy number (CN)-low-like and CN-high-like ECs with an area under the receiver operating characteristic curve (AUROC) of 0.78 (95% CI 0.58–0.91). The model further differentiated TMB-H from TMB-low (TMB-L) tumors with an AUROC of 0.87 (95% CI 0.73–0.95). Peritumoral-rim radiomic features were most relevant to both classifications (p ≤ 0.044). Radiomic analysis achieved moderate accuracy in identifying MMR-D and TMB-H ECs directly from CE-CT. Radiomics may provide an adjunct tool to molecular profiling, especially given its potential advantage in the setting of intratumor heterogeneity.

## Introduction

Medical imaging plays an essential role in oncology care during all stages of clinical management. Until recently, the practice of radiology was based largely on subjective image interpretation by human observers. Radiomics is a rapidly developing field that robustly processes and converts radiologic images into minable quantitative data^1^. Radiomic approaches that implement machine learning algorithms have already successfully uncovered a number of associations between radiomic signatures and key genomic drivers across several cancer types^2–5^. This growing body of literature points to a latent opportunity to integrate imaging as a biomarker for new targeted therapies^6–9^.

Endometrial cancer (EC) is the most frequent gynecologic cancer in the United States^10^. Traditionally, tumor histology, grade, and International Federation of Gynecology and Obstetrics (FIGO) stage are the key prognostic factors^11,12^. However, the FIGO system has limited prognostic relevance in uterine-confined disease^13^, and there is interobserver-variability both in the pathologic assessment of tumor grade and in the diagnosis of high-grade ECs^14–17^. Beyond this histologic classification, The Cancer Genome Atlas (TCGA) has defined four molecular subtypes of EC that correlate with prognosis: DNA polymerase epsilon (*POLE*) mutant [ultramutated], microsatellite instability-high (MSI-H)/mismatch-repair deficient (MMR-D) [hypermutated], copy-number low (CN-low) [endometrioid-like], and copy-number high (CN-high) [serous-like]^18^. The above molecular classification combined with traditional prognostic factors is now advocated as the preferred schema to risk-stratify EC, design prospective clinical trials, and potentially personalize patient therapy^19^.

Accurate molecular classification is essential given the Food and Drug Administration (FDA) approval of Pembrolizumab, an anti-programmed cell death 1 (anti-PD-1) antibody, for treatment of MMR-D/MSI-H solid tumors^20–23^. Beyond MMR status, the determination of tumor mutational burden (TMB) is also potentially relevant given the reports of response to immune checkpoint blockade in patients with *POLE*-mutated EC^24^, as well as the ongoing prospective clinical trials evaluating PD-1/PD-L1 monotherapy in TMB-high (TMB-H) solid tumors (Clinicaltrials.gov, NCT02091141, NCT03668119)^25^.

We hypothesize that medical imaging, processed with radiomics, can uncover the salient associations with the underlying tumor biology. If these associations are confirmed, radiomic analysis may serve as a surrogate for molecular profiling in the settings when tissue is not available for immunohistochemical or mutational analysis, or if the results are ambiguous. Moreover, at present, therapeutic decisions are based largely on the analysis of primary tumors, and may miss MMR-D that is present only in the advanced/recurrent setting^26,27^. Radiomic analysis has the potential to simultaneously and non-invasively assess both primary and metastatic lesions, potentially capturing inter-lesion molecular heterogeneity.

In this study, we aimed to evaluate whether radiomic analysis of contrast-enhanced computed tomography (CE-CT) obtained during initial staging of EC can identify MMR-D and TMB-H tumors. Two key considerations that motivated our focus on preoperative CE-CT were: (1) the recommendations by the National Comprehensive Cancer Network (NCCN) guidelines to obtain CE-CT for the initial staging of high-grade EC and restaging of suspected recurrence or metastasis^28^ and (2) sequencing and immunohistochemical analysis were performed on primary tumors.

## Methods

### Overview

**Figure 1:**
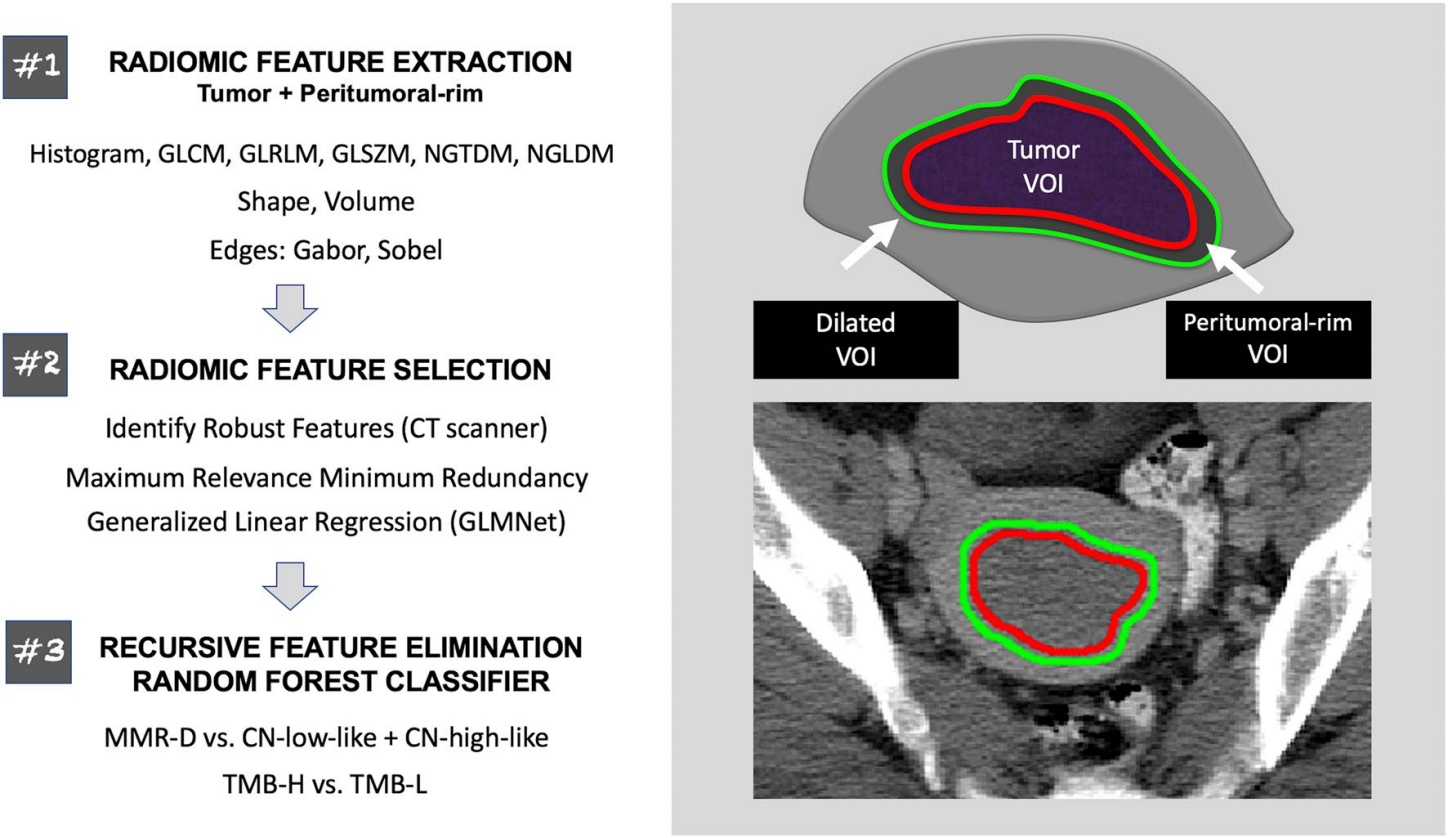
Schematic overview of the methods employed in this study. The radiologist-defined tumor VOIs were used to extract intra-tumoral radiomic features. Peritumoral-rim was generated by automatically expanding the tumor contours by 3 mm and subtracting the dilated VOI from the tumor VOI. Peritumoral-rim features were computed as the differences in the values of radiomic features between the dilated VOI and the tumor VOI. The features are later down-selected via a multi-step approach; the selected features were z-score standardized and used to construct the classifiers. *CT* computed tomography, *GLCM* gray level co-occurrence matrix, *GLRLM* gray level run length matrix, *GLSZM* gray level size zone matrix, *NGTDM* neighborhood gray tone difference matrix, *NGLDM* neighborhood gray level dependence matrix, *MMR-D* DNA mismatch repair-deficient, *CN* copy number, *TMB-H* tumor mutational burden-high, *TMB-L* tumor mutational burden-low, *VOI* volume of interest.

Machine learning classifiers comprised of radiomic features and clinical variables (patient age, tumor histology, grade, and FIGO stage) were trained to (1) distinguish MMR-D from CN-low-like and CN-high-like ECs and (2) identify TMB-H tumors (Fig. 1). Classification models were built using the training dataset and then validated on the holdout test dataset. The Institutional Review Board (IRB) of Memorial Sloan Kettering Cancer Center (MSKCC) approved this retrospective Health Insurance Portability and Accountability Act-compliant study and waived the requirement for informed consent. All subjects included in this study had previously consented to an IRB-approved study of targeted massively parallel sequencing in patients with solid tumors (Memorial Sloan Kettering-Integrated Mutation Profiling of Actionable Cancer Targets, MSK-IMPACT; NCT01775072)^29^. All methods were carried out in accordance with relevant guidelines and regulations.

### Eligibility criteria

**Figure 2:**
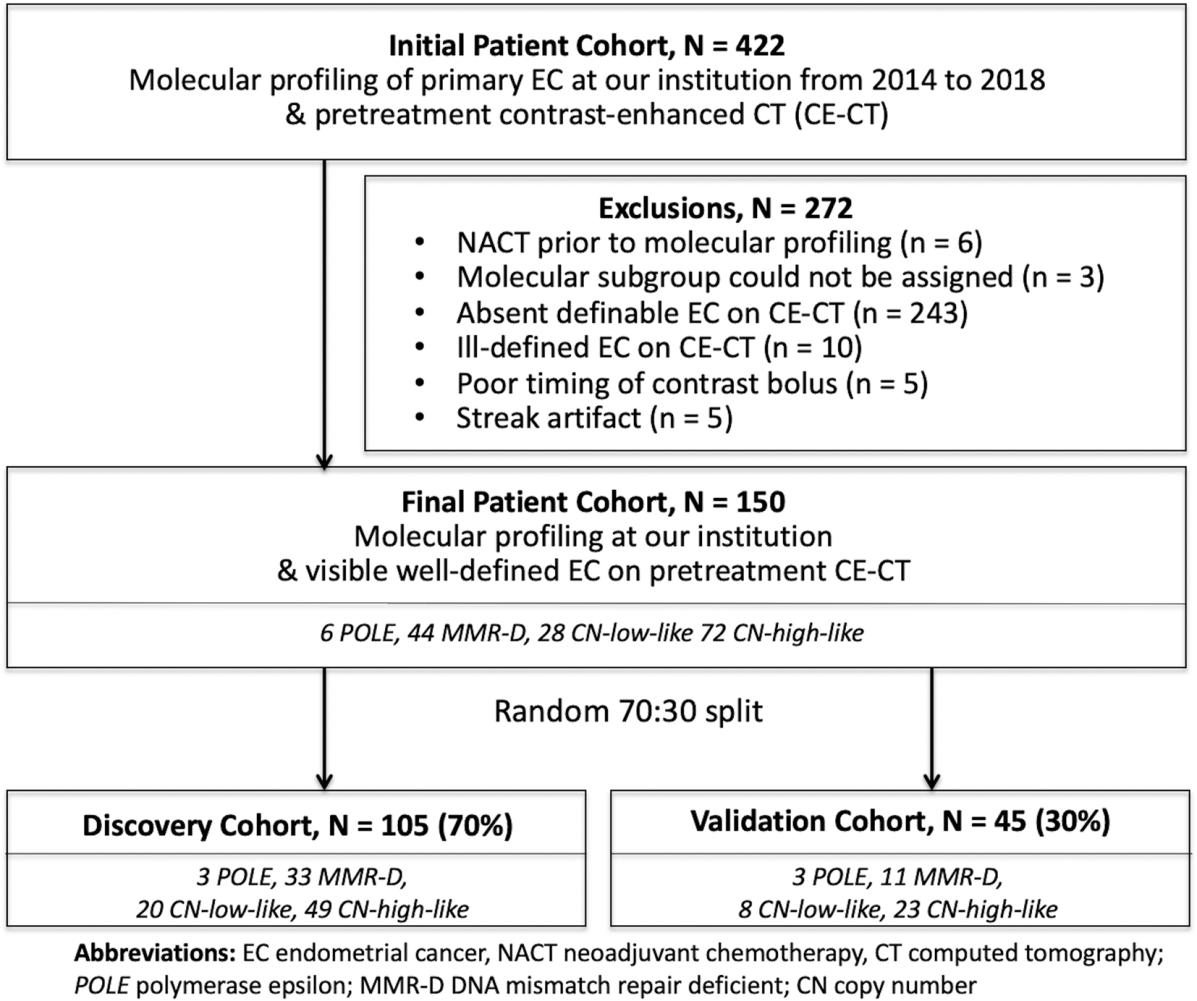
Flow chart illustrating the patient selection criteria. *EC* endometrial cancer, *CE-CT* contrast-enhanced computed tomography, *NACT* neoadjuvant chemotherapy, *POLE* polymerase epsilon, *MMR-D* DNA mismatch repair-deficient, *CN* copy number.

Our institutional database was queried to identify all patients who met the following eligibility criteria: (1) molecular profiling of primary ECs with MSK-IMPACT sequencing and immunohistochemical analysis of p53 and MMR proteins between 2014 and 2018 and (2) preoperative CE-CT with definable endometrial tumor (Fig. 2). Patients with all histologic subtypes of EC and all FIGO stages were included if they otherwise met the eligibility criteria. The final study population included 150 patients; this cohort was randomly split into the discovery (n = 105, 70%) and validation (n = 45, 30%) groups (Table 1, Fig. 2). Electronic medical records were reviewed for pertinent clinical information.

**Table 1:** Patient characteristics.

	Entire cohort N = 150	Discovery cohort N = 105	Validation cohort N = 45	p-values*
**Median patient age, years (IQR)**	64 (58–71)	65 (59–71)	62 (56–68)	0.10
**Histology, number (%)**				0.09
Endometrioid	62 (41.3%)	45 (42.9%)	17 (37.8%)	
Serous	31 (20.7%)	22 (21.0%)	9 (20.0%)	
Clear cell	11 (7.3%)	8 (7.6%)	3 (6.7%)	
Carcinosarcoma	26 (17.3%)	15 (14.3%)	11 (24.4%)	
Undifferentiated/dedifferentiated	6 (4.0%)	2 (1.9%)	4 (8.9%)	
Unclassified high-grade type	14 (9.3%)	13 (12.4%)	1 (2.2%)	
**Tumor grade, number (%)**				0.60
Well/moderately differentiated	109 (72.7%)	75 (71.4%)	34 (75.6%)	
Poorly differentiated	41 (27.3%)	30 (28.6%)	11 (24.4%)	
**Stage, number (%)**				0.80
Extra-uterine	84 (56.0%)	58 (55.2%)	26 (57.8%)	
Uterine-confined	66 (44.0%)	47 (44.8%)	19 (42.2%)	
**Molecular subtype, number (%)**				0.59
*POLE*	6 (4.0%)	3 (2.9%)	3 (6.7%)	
MMR-D	44 (29.3%)	33 (31.4%)	11 (24.4%)	
CN-low-like (endometrioid-like)	28 (18.7%)	20 (19.0%)	8 (17.8%)	
CN-high-like (serous-like)	72 (48.0%)	49 (46.7%)	23 (51.1%)	
**Median TMB, mut/Mb (IQR)**	6.7 (3.6, 24.4)	6.7 (3.9, 25.7)	6.9 (3.5, 15.8)	0.40

*IQR* interquartile range, *FIGO* the International Federation of Gynecology and Obstetrics, *POLE* polymerase epsilon, *MMR-D* DNA mismatch repair-deficient, *CN* copy number, *TMB* tumor mutational burden, *mut/Mb* mutations per megabase.

*Categorical variables were compared using Fisher exact test; continuous variables were compared with Mann–Whitney U-test.

### Molecular profiling and classification of ECs

Tumor and matched normal DNA were subjected to MSK-IMPACT sequencing, a Food and Drug Administration (FDA)-approved, Clinical Laboratory Improvement Amendments (CLIA)-certified, massively parallel sequencing platform to identify somatic and germline genetic alterations in up to 468 cancer-related genes^29,30^. A clinical algorithm was applied based on DNA polymerase epsilon (*POLE*) exonuclease domain mutations identified by sequencing and immunohistochemistry (IHC) for p53 and MMR proteins to stratify ECs into *POLE*, MMR-deficient (MMR-D), CN-low-like, and CN-high-like subtypes. These subtypes are analogous but not identical to the TCGA subtypes^14,31,32^. IHC for p53 and DNA mismatch repair proteins (MSH2, MSH6, MLH1, and PMS2) was performed as previously described^33,34^. The MSK-IMPACT and IHC data for all study patients were reviewed by a board-certified gynecologic pathologist (D.F.D.).

TMB was computed as the number of nonsynonymous somatic mutations-per-megabase (mut/Mb) using MSK-IMPACT sequencing, targeting exons and selected introns of up to 468 cancer-related genes^29,30^. A universal, pan-solid tumor TMB cutoff to distinguish responders from non-responders to immune checkpoint inhibitors is yet to be defined. We selected a cut-point of > 15.5 mutations/Mb (i.e. ≥ 16 mut/Mb when rounded up to the nearest whole number). This is a clinically meaningful cut-point that mirrors the enrollment criteria of the MyPathway (NCT02091141), a study of Atezolizumab that is actively enrolling patients with TMB-H advanced solid tumors.

### Segmentation and image preprocessing

Details of CE-CT acquisition are provided in the Supplement. Two radiologists (Y.L. and J.N.) with expertise and experience in gynecologic oncologic imaging manually contoured all tumors in consensus. The radiologists used the Insight Segmentation and Registration Toolkit-Segmentation platform (ITK-SNAP) version 3.6 (https://itksnap.org) to trace the outer margin of each lesion on every tumor-containing image, a process that resulted in radiologist-defined tumor volumes-of-interest (VOI)^35^. To ensure consistent extraction of texture features, all CT images and corresponding VOIs were resampled to the uniform voxel size of 1 × 1 × 1 mm^3^ using the in-house software wrapper written in C++ around the Insight ToolKit software library^36^.

In addition to tumor VOI, the peritumoral-rim was generated for each lesion/patient to capture the invasive edge of the tumor and interrogate the environment surrounding the tumor. First, dilated VOI was produced via automated expansion of the tumor contour by 3 mm. Then, tumor VOI was subtracted from the dilated VOI, resulting in the peritumoral-rim (Fig. 1).

### Radiomic feature extraction

Radiomic features were computed using the open-source Computational Environment for Radiological Research (CERR) software (https://github.com/cerr/CERR/) and according to the standards of the image biomarker standardization initiative (IBSI)^37–39^. For each patient, the radiomic features were extracted both from within the tumor and from the dilated VOI; peritumoral-rim features were then computed as a difference in the values of features between the dilated VOI and the tumor VOI. Two hundred (100 intra-tumoral and 100 peritumoral-rim) radiomic features were generated for each lesion/patient as detailed in the Supplement.

### Radiomic feature selection

Dimensionality reduction was accomplished via a multi-step approach. First, the Kruskal–Wallis test was used to identify and remove all unstable radiomic features, i.e., features with significant variations across CT scanner manufacturers. Next, the maximum relevance and minimum redundancy (mRMR) method was used to select the top-80% most relevant and least redundant features^40^.

Lastly, mRMR selected features were passed through an additional pre-filtering step using the generalized linear regression (GLMNet)^41,42^. GLMNet was trained with repeated five-fold cross validation, ten repetitions, and up-sampling to handle class imbalance. At the end, seventy features with the importance of > 0 were selected to build radiomic-clinical classification models; all features were z-score standardized (scaled and centered to a mean of 0 and standard deviation of 1.0).

### Integrated radiomic-clinical classification models

Recursive feature elimination random forest classifiers (RFE-RF) with 2000 decision trees and repeated (n = 10) five-fold nested internal cross-validation were built on the data from the training dataset (discovery cohort; n = 105)^43,44^. All features were ranked according to their importance in the classification using Gini feature importance^44^. Gini feature importance measures the overall probability of misclassification when using a given feature in the individual trees of the RF classifier^43^. Features with Gini feature importance of > 25.0 (i.e., features selected > 10% of the time in the 250 analysis folds) were considered relevant to the classification. Cross-validation accuracy was computed using the cases that were not included in the individual analysis folds. Integrated radiomic-clinical classification models with the best performance among the cross-validation folds were selected for the validation on the holdout test dataset (validation cohort; n = 45).

### Statistical analysis

The models were evaluated with an area under the receiver operating characteristic curve (AUROC). Sensitivity, specificity, positive predictive value (PPV), and negative predictive value (NPV) including 95% confidence intervals (CI) were reported. The models were compared with the deLong test^45^.

The univariate associations between the relevant features selected by the RFE-RF and outcomes (distinguishing MMR-D from CN-low-like and CN-high-like ECs; identifying TMB-H tumors) were examined with the Mann–Whitney U-test. The Benjamini–Hochberg method was used to correct for multiple comparisons^46^. All statistical analyses were performed using R software, version 3.3.3^47^.

## Results

### Patients

Patient characteristics are summarized in Table 1. Median patient age at EC diagnosis was 64 years (inter-quartile range (IQR) 58–71 years). Endometrioid adenocarcinoma (62/150; 41.3%), serous carcinoma (31/150; 20.7%) and carcinosarcoma (26/150; 17.3%) were the most prevalent histologic subtypes. The distribution of the molecular subtypes was as follows: 6/150 (4%) *POLE*, 44/150 (29.3%) MMR-D, 28/150 (18.7%) CN-low-like, and 72/150 (48%) CN-high-like. All *POLE* and 41/44 (93%) MMR-D tumors were TMB-H (i.e. > 15.5 mutations/Mb); all CN-low-like and all CN-high-like tumors were TMB-L. Two of three MMR-D tumors with TMB-L had low tumor cell content (i.e. ≤ 20%). The discovery (n = 105) and validation (n = 45) groups were similar with respect to the clinical variables, molecular subtypes, and TMB status (p ≥ 0.05 all comparisons) (Table 1).

### Integrated radiomic-clinical machine learning classifier can identify MMR-D tumors

**Figure 3:**
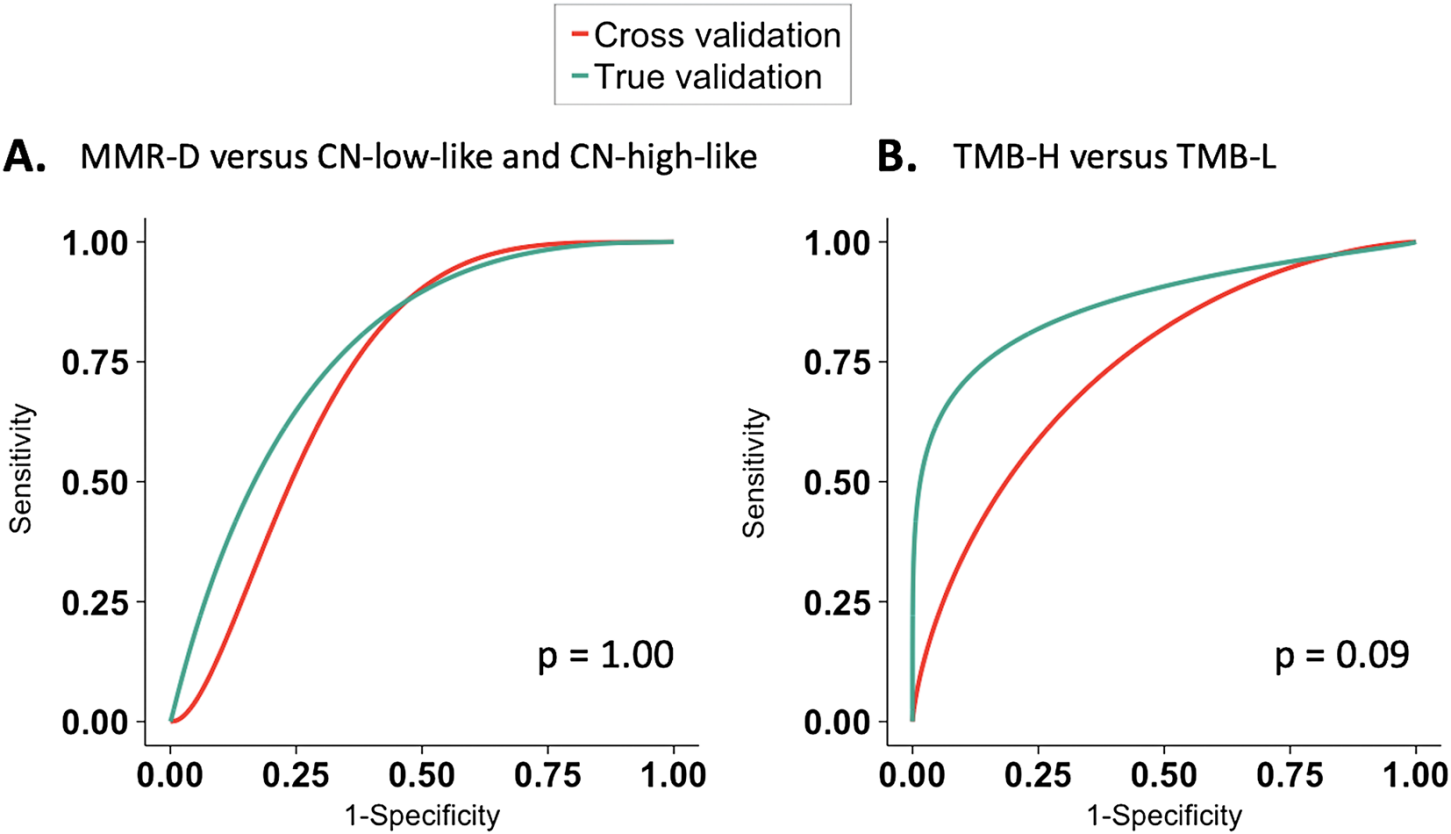
Receiver operator characteristic curves demonstrate the performance of the integrated clinical-radiomic models in the training dataset and the holdout test dataset to (**A**) differentiate MMR-D from CN-low-like and CN-high-like tumors, and (**B**) distinguish TMB-H from TMB-L ECs. *EC* endometrial cancer, *MMR-D* DNA mismatch repair-deficient, *CN* copy number, *TMB* tumor mutational burden.

The performance of the radiomic-clinical model to distinguish MMR-D from CN-low-like and CN-high-like ECs is summarized in Table 2. For this analysis, ECs of *POLE* molecular subtype were excluded. We found that our model achieved a cross-validation AUROC of 0.78 (95% CI 0.67–0.88) for the training dataset and a true validation AUROC of 0.78 (95% CI 0.58–0.91) for the test dataset (p = 1.0) (Fig. 3A).

**Table 2:** The perfromance of the integrated radiomic-clinical classification model to differentiate MMR-D from CN-low-like and CN-high-like ECs.

Data sets	AUROC (95% CI)	Sensitivity (95% CI)	Specificity (95% CI)	PPV (95% CI)	NPV (95% CI)	p-value^*^
Training set (discovery cohort, N = 102^b^)	0.78^a^ (0.67, 0.88)	0.67 (0.48, 0.82)	0.77 (0.65, 0.86)	0.58 (0.41, 0.74)	0.83 (0.71, 0.91)	1.0
Test set (validation cohort, N = 42^b^)	0.78 (0.58, 0.91)	0.64 (0.31, 0.89)	0.74 (0.55, 0.88)	0.47 (0.21, 0.73)	0.85 (0.66, 0.96)	

*EC* endometrial cancer, *AUROC* area under the receiver operating curve, *CI* confidence interval, *PPV* positive predictive value, *NPV* negative predictive value, *MMR-D* DNA mismatch repair-deficient, *CN* copy number.

^*^deLong test.

^a^Cross-validated AUROC.

^b^Patients with polymerase epsilon (POLE) subtypes of EC were excluded from this analysis.

The radiomic-clinical model identified three peritumoral-rim Gabor edge features as the most relevant features to the classification, i.e., Gini feature importance > 25.0 (Supplemental Table 1). The above peritumoral-rim features remained significant after the adjustment for multiple comparisons (p ≤ 0.008) (Supplemental Table 1). Peritumoral-rim radiomics interrogates and potentially quantifies the heterogeneity across the interface between the periphery of tumor and its environment; Gabor filters are the edge detectors that capture the orientation-sensitive information. Our results suggest that the peritumoral-rim of MMR-D ECs demonstrates higher heterogeneity compared to the peritumoral-rim of CN-low-like and CN-high-like tumors.

**Table 3:** The performance of the integrated radiomic-clinical classification model to distinguish TMB-high from TMB-low ECs.

Data sets	AUROC (95% CI)	Sensitivity (95% CI)	Specificity (95% CI)	PPV (95% CI)	NPV (95% CI)	p-value^*^
Training set (discovery cohort, N = 105	0.74^a^ (0.64, 0.84)	0.83 (0.66, 0.93)	0.73 (0.61, 0.83)	0.60 (0.45, 0.74)	0.89 (0.78, 0.96)	0.09
Test set (validation cohort, N = 45)	0.87 (0.73, 0.95)	0.75 (0.43, 0.95)	0.82 (0.65, 0.93)	0.60 (0.32, 0.84)	0.90 (0.73, 0.98)	

*TMB* tumor mutational burden, *EC* endometrial cancer, *AUROC* Area under the receiver operating curve, *CI* confidence interval, *PPV* positive predictive value, *NPV* negative predictive value.

*deLong test.

^a^Cross-validated AUROC.

### Integrated radiomic-clinical machine learning classifier can identify TMB-H tumors

As a next step, we assessed the performance of the radiomic-clinical model to differentiate all TMB-H ECs, including ECs of MMR-D and *POLE* subtypes, from TMB-L tumors (Table 3). We found that our model achieved a cross-validation AUROC of 0.74 (95% CI 0.64–0.84) for the training dataset and a true validation AUROC of 0.87 (95% CI 0.73–0.95) for the test dataset (p = 0.09) (Fig. 3B).

The radiomic-clinical model selected 21 features as the most important to the classification, i.e., Gini feature importance > 25.0 (Supplemental Table 2). Of these, 12 features remained significantly associated with TMB status after the adjustment for multiple comparisons (p ≤ 0.044). Again, the peritumoral-rim features including Gabor edge features were among the most relevant to the classification. Concretely, the peritumoral-rim features accounted for 9/12 (75%) significant features for identifying TMB-H tumors (Supplemental Table 2).

## Discussion

Here, we demonstrate that CE-CT radiomics can distinguish MMR-D from CN-low-like and CN-high-like ECs and identify TMB-H ECs. Our machine learning classifier was built using clinical variables and both intra-tumoral and peritumoral-rim radiomic features; the model was subsequently validated on the holdout test dataset. The resultant integrated clinical-radiomic models achieved true validation AUROCs of 0.78 and 0.87 for classifying MMR-D and identifying TMB-H ECs, respectively. MMR IHC is a highly sensitive and specific tool to determine MMR status, however, the screening is not universal beyond tertiary centers^48^. In view of the common use of CE-CT to stage ECs at the time of initial diagnosis and recurrence, non-invasive radiomic-based identification of MMR-D and TMB-H tumors may reduce the cost and improve the accessibility of molecular stratification. Further, radiomics may complement the evaluation of TMB status in the setting of low tumor content, a scenario that is not uncommon with primary low-grade endometrioid ECs.

In our study, the machine learning classifier selected peritumoral-rim radiomics as the most relevant to the classification. This finding may suggest that MMR-D and TMB-H ECs demonstrate greater heterogeneity at the interface between the tumor and its environment when compared to CN-low-like and CN-high-like/TMB-L tumors, respectively. A number of studies across multiple tumor types have reported on the value of peritumoral-rim radiomics (in addition to or beyond that of intra-tumor radiomics) in uncovering associations with tumor biology, prognosis, and treatment response^8,49–54^. The peritumoral-rim encompasses the invasive edge of the tumor and, thus, potentially provides insight into the role of the tumor microenvironment in cancer biology and behavior. MMR-D tumors are known to have a higher composition of tumor infiltrating lymphocytes (TILs) compared to MMR-proficient disease^55,56^. In breast cancer, several studies have demonstrated an association between peritumoral features and TIL density at the tumor margins^8,51^. This is a rich area of research; further studies are warranted to explore the associations between the peritumoral-rim radiomics and the density of peritumoral TIL in EC.

To our knowledge, only two recent studies evaluated whether machine learning can identify MMR-D/MSI-H tumors, focusing on gastrointestinal tumors^57,58^. Kather et al*.* implemented automated tumor detection on the Hematoxylin and Eosin (H&E)-stained sections of colorectal and gastric cancers and tessellated each lesion into the color-normalized tiles. Using the color tiles, a deep learning model was trained on TCGA cohorts to identify MSI-H tumors, achieving AUCs of 0.77–0.84 depending on the cohort^57^. The model was then validated on a multi-institutional cohort with a true validation AUC of 0.84. Interestingly, the authors found that MSI was associated spatially with poor differentiation and lymphocyte-rich tumor areas, consistent with prior histopathology literature. Golia Pernicka et al*.* evaluated whether CE-CT-based radiomics can predict MMR-D status in patients with colon cancer^58^. Similar to our study, the combined clinical-radiomic model predicted MMR-D colon cancer with an AUC of 0.80 for the training dataset and 0.79 for the test dataset. The combined model was slightly superior to the clinical-only (AUC of 0.74) and radiomic-only models (AUC of 0.76). Peritumoral-rim features were not included in the analysis.

To date, only one prior study has explored the role of radiomics in decoding TMB. Wang et al*.* evaluated 61 tumors in 51 patients with surgically proven early stage lung cancer and preoperative CT. The authors reported an AUC of 0.671 for predicting TMB status using a combined clinical-radiomic model^59^. Comparison between Wang et al*.* and our findings is limited by the differences in tumor types and the lack of universal definition of TMB-H status.

ECs are a heterogenous group of tumors with diverse clinical behaviors and distinct genomic alterations. Conventional chemotherapy^60,61^ and endocrine treatments^62,63^ have limited efficacy in advanced or recurrent disease, highlighting the need for novel therapeutic paradigms^64^. The molecular stratification of ECs may expand further our understanding of tumor behavior and response to therapy, and, thus, accelerates the realization of precision medicine through better patient selection and innovative clinical trial design. Nevertheless, the widespread implementation of genomic profiling and molecular classification is limited by the cost, as well as availability of sequencing, tissue availability/tissue purity, functional assays, and area-specific expertise. Furthermore, MMR IHC is frequently performed on the primary tumor, which may miss acquired MMR deficiency in metastatic/recurrent disease^26,27^. Reliable and cost-effective identification of patients with MMR-D/MSI-H ECs are essential given the FDA approval of Pembrolizumab for MMR-D/ MSI-H solid tumors^22^. Robust and inexpensive assessment of TMB status is also timely given the ongoing prospective clinical trials which are enrolling patients with pan-solid tumors on the basis of TMB and other molecular classifiers (NCT03668119, NCT0291141)^64^. Our findings suggest the potential of radiomics as an adjunct tool to molecular profiling, especially given its possible advantage in the presence of intra-tumor heterogeneity.

Our study has several limitations, including retrospective study design, relatively small patient cohort, and the fact that all patients were sourced from a single institution. Notably, many patients in our cohort (36%) were imaged at various outside institutions, suggesting that our models may be generalizable across multiple CT protocols and vendors. Further validation of this approach utilizing large multi-institutional cohorts will be essential to exclude overfitting and confirm the applicability of our results across different institutions/patient populations. In addition, many ECs were challenging to delineate on CE-CT, probably due to smaller size and ill-defined borders. This limitation may be overcome with Magnetic Resonance Imaging (MRI), which has superior soft tissue contrast, and, according to the NCCN guidelines, is considered for evaluating local disease extent during initial work-up^28^. However, preoperative MRI may be unavailable for many patients because of the growing shift toward sentinel lymph node sampling/mapping (making preoperative information about the depth of myometrial invasion less critical)^11^. The radiomic analysis may be enhanced further by utilizing deep learning algorithms. Deep learning identifies salient features without the need for segmentation and, given enough training data, outperforms traditional machine learning approaches that are based on “hand-crafted” features^65^. Finally, our study was restricted to primary ECs given that the sequencing and IHC analyses was performed on primary tumors. Considering that pembrolizumab is currently FDA-approved in the advanced or recurrent setting, it will be important to validate our results in the advanced disease setting.

In conclusion, we have developed clinical-radiomic machine learning models to non-invasively identify MMR-D and TMB-H ECs from CE-CT images. This serves as a proof-of-principle that radiomic models should be investigated and validated further in large multi-institutional cohorts with the aim of developing these algorithms as a reproducible companion/complementary diagnostic for clinical trial enrollment and standard-of-care treatment^66^. Moving forward, it will be also informative to deploy radiomics to capture intra-patient variability and to decode other key EC genomic drivers with clinical indications.

## Supplementary information

Supplementary Information.

## Data Availability

The data and the R code that support the findings of this study are available at https://github.com/harveerar/SciRepEndometrial2020.

## References

[1] Gillies RJ, Kinahan PE, Hricak H (2016). Radiomics: Images are more than pictures, they are data. Radiology.

[2] Zhou M (2018). Non-small cell lung cancer radiogenomics map identifies relationships between molecular and imaging phenotypes with prognostic implications. Radiology.

[3] Gevaert O (2014). Glioblastoma multiforme: Exploratory radiogenomic analysis by using quantitative image features. Radiology.

[4] Sutton EJ (2016). Breast cancer molecular subtype classifier that incorporates MRI features. J. Magnet. Resonance Imaging JMRI.

[5] Grossmann P (2017). Defining the biological basis of radiomic phenotypes in lung cancer. Elife..

[6] Sun R (2018). A radiomics approach to assess tumour-infiltrating CD8 cells and response to anti-PD-1 or anti-PD-L1 immunotherapy: An imaging biomarker, retrospective multicohort study. Lancet Oncol..

[7] Trebeschi S (2019). Predicting response to cancer immunotherapy using noninvasive radiomic biomarkers. Ann. Oncol Off. J. Eur. Soc. Med. Oncol. ESMO.

[8] Braman N (2019). Association of peritumoral radiomics with tumor biology and pathologic response to preoperative targeted therapy for HER2 (ERBB2)-positive breast cancer. JAMA Netw. Open.

[9] Himoto Y (2019). Computed tomography-derived radiomic metrics can identify responders to immunotherapy in ovarian cancer. JCO Precis. Oncol..

[10] Siegel RL, Miller KD, Jemal A (2019). Cancer statistics, 2019. CA Cancer J. Clin..

[11] Colombo N (2016). ESMO-ESGO-ESTRO consensus conference on endometrial cancer: Diagnosis, treatment and follow-up. Int. J. Gynecol. Cancer Off. J. Int. Gynecol. Cancer Society.

[12] Singh N (2019). Pathologic prognostic factors in endometrial carcinoma (other than tumor type and grade). Int. J. Gynecol. Pathol. Off. J. Int. Society Gynecol. Pathol..

[13] Pecorelli S (2009). Revised FIGO staging for carcinoma of the vulva, cervix, and endometrium. Int. J. Gynaecol. Obstetr. Off. Organ Int. Federation Gynaecol. Obstetr..

[14] Murali R, Delair DF, Bean SM, Abu-Rustum NR, Soslow RA (2018). Evolving roles of histologic evaluation and molecular/genomic profiling in the management of endometrial cancer. J. Natl. Comprehensive Cancer Netw. JNCCN.

[15] Gilks CB, Oliva E, Soslow RA (2013). Poor interobserver reproducibility in the diagnosis of high-grade endometrial carcinoma. Am. J. Surg. Pathol..

[16] Soslow RA (2010). Endometrial carcinomas with ambiguous features. Semin. Diagn. Pathol..

[17] Bendifallah S (2015). Just how accurate are the major risk stratification systems for early-stage endometrial cancer?. Br. J. Cancer.

[18] Kandoth C (2013). Integrated genomic characterization of endometrial carcinoma. Nature.

[19] Murali R, Soslow RA, Weigelt B (2014). Classification of endometrial carcinoma: more than two types. Lancet Oncol..

[20] Le DT (2015). PD-1 blockade in tumors with mismatch-repair deficiency. N. Engl. J. Med..

[21] Makker V (2017). New therapies for advanced, recurrent, and metastatic endometrial cancers. Gynecol. Oncol. Res. Practice.

[22] Marcus L, Lemery SJ, Keegan P, Pazdur R (2019). FDA approval summary: Pembrolizumab for the treatment of microsatellite instability-high solid tumors. Clin. Cancer Res. Off. J. Am. Assoc. Cancer Res..

[23] Marabelle, A. *et al.* Efficacy of pembrolizumab in patients with noncolorectal high microsatellite instability/mismatch repair–deficient cancer: Results from the phase II KEYNOTE-158 study. **38**, 1–10. 10.1200/jco.19.02105 (2020). 10.1200/JCO.19.02105 PMC8184060 31682550

[24] Wang F (2019). Evaluation of POLE and POLD1 mutations as biomarkers for immunotherapy outcomes across multiple cancer types. JAMA Oncol..

[25] Chan TA (2019). Development of tumor mutation burden as an immunotherapy biomarker: Utility for the oncology clinic. Ann. Oncol. Off. J. Eur. Society Med. Oncol. ESMO.

[26] Ashley CW (2019). Analysis of mutational signatures in primary and metastatic endometrial cancer reveals distinct patterns of DNA repair defects and shifts during tumor progression. Gynecol. Oncol..

[27] Ta RM, Hecht JL, Lin DI (2018). Discordant loss of mismatch repair proteins in advanced endometrial endometrioid carcinoma compared to paired primary uterine tumors. Gynecol. Oncol..

[28] Koh WJ (2018). Uterine neoplasms, version 1.2018, NCCN clinical practice guidelines in oncology. J. Natl. Comprehensive Cancer Netw. JNCCN.

[29] Cheng DT (2015). Memorial sloan kettering-integrated mutation profiling of actionable cancer targets (MSK-IMPACT): A hybridization capture-based next-generation sequencing clinical assay for solid tumor molecular oncology. J. Mol. Diagnostics JMD.

[30] Zehir A (2017). Mutational landscape of metastatic cancer revealed from prospective clinical sequencing of 10,000 patients. Nat. Med..

[31] Talhouk A (2015). A clinically applicable molecular-based classification for endometrial cancers. Br. J. Cancer.

[32] DeLair DF (2017). The genetic landscape of endometrial clear cell carcinomas. J. Pathol..

[33] Garg K (2009). Selection of endometrial carcinomas for DNA mismatch repair protein immunohistochemistry using patient age and tumor morphology enhances detection of mismatch repair abnormalities. Am. J. Surg. Pathol..

[34] Garg K (2010). p53 overexpression in morphologically ambiguous endometrial carcinomas correlates with adverse clinical outcomes. Modern Pathol. Off. J. US Can. Acad. Pathol. Inc..

[35] Yushkevich PA (2006). User-guided 3D active contour segmentation of anatomical structures: Significantly improved efficiency and reliability. NeuroImage.

[36] Yoo TS (2002). Engineering and algorithm design for an image processing Api: A technical report on ITK—The Insight Toolkit. Stud. Health Technol. Inform..

[37] Zwanenburg A (2020). The image biomarker standardization initiative: standardized quantitative radiomics for high-throughput image-based phenotyping. Radiology.

[38] Apte AP (2018). Technical Note: Extension of CERR for computational radiomics: A comprehensive MATLAB platform for reproducible radiomics research. Med. Phys..

[39] Deasy JO, Blanco AI, Clark VH (2003). CERR: A computational environment for radiotherapy research. Med. Phys..

[40] Peng H, Long F, Ding C (2005). Feature selection based on mutual information: Criteria of max-dependency, max-relevance, and min-redundancy. IEEE Trans. Pattern Anal. Mach. Intell..

[41] Friedman J, Hastie T, Tibshirani R (2010). Regularization paths for generalized linear models via coordinate descent. J. Stat. Softw..

[42] Simon, N., Friedman, J. H., Hastie, T. & Tibshirani, R. Regularization paths for Cox's proportional hazards model via coordinate descent. **39**, 13. 10.18637/jss.v039.i05 (2011). 10.18637/jss.v039.i05 PMC4824408 27065756

[43] Breiman L (2001). Random forests. Mach. Learn..

[44] Fehr D (2015). Automatic classification of prostate cancer Gleason scores from multiparametric magnetic resonance images. Proc. Natl. Acad. Sci. USA.

[45] DeLong ER, DeLong DM, Clarke-Pearson DL (1988). Comparing the areas under two or more correlated receiver operating characteristic curves: A nonparametric approach. Biometrics.

[46] Holm S (1979). A simple sequentially rejective multiple test procedure. Scand. J. Stat..

[47] R Core Team. *R: A language and environment for statistical computing* [Internet]. Vienna, Austria. https://www.R-project.org/ (2020).

[48] Eriksson J (2019). Mismatch repair/microsatellite instability testing practices among US physicians treating patients with advanced/metastatic colorectal cancer. J. Clin. Med..

[49] Dou TH, Coroller TP, van Griethuysen JJM, Mak RH, Aerts H (2018). Peritumoral radiomics features predict distant metastasis in locally advanced NSCLC. PLoS ONE.

[50] Khorrami M (2019). Predicting pathologic response to neoadjuvant chemoradiation in resectable stage III non-small cell lung cancer patients using computed tomography radiomic features. Lung Cancer.

[51] Braman NM (2017). Intratumoral and peritumoral radiomics for the pretreatment prediction of pathological complete response to neoadjuvant chemotherapy based on breast DCE-MRI. Breast Cancer Res..

[52] Beig N (2019). Perinodular and intranodular radiomic features on lung CT images distinguish adenocarcinomas from granulomas. Radiology.

[53] Prasanna P, Patel J, Partovi S, Madabhushi A, Tiwari P (2017). Radiomic features from the peritumoral brain parenchyma on treatment-naive multi-parametric MR imaging predict long versus short-term survival in glioblastoma multiforme: Preliminary findings. Eur. Radiol..

[54] Xu X (2019). Radiomic analysis of contrast-enhanced CT predicts microvascular invasion and outcome in hepatocellular carcinoma. J. Hepatol..

[55] Pakish JB (2017). Immune microenvironment in microsatellite-instable endometrial cancers: Hereditary or sporadic origin matters. Clin. Cancer Res. Off. J. Am. Assoc. Cancer Res..

[56] Narayanan S (2019). Tumor infiltrating lymphocytes and macrophages improve survival in microsatellite unstable colorectal cancer. Sci. Rep..

[57] Kather JN (2019). Deep learning can predict microsatellite instability directly from histology in gastrointestinal cancer. Nat. Med..

[58] Pernicka JSG, Gagniere J, Chakraborty J (2019). Radiomics-based prediction of microsatellite instability in colorectal cancer at initial computed tomography evaluation. Abdom. Radiol..

[59] Wang X (2019). Decoding tumor mutation burden and driver mutations in early stage lung adenocarcinoma using CT-based radiomics signature. Thorac. Cancer.

[60] Thigpen JT, Buchsbaum HJ, Mangan C, Blessing JA (1979). Phase II trial of adriamycin in the treatment of advanced or recurrent endometrial carcinoma: A Gynecologic Oncology Group study. Cancer Treat. Rep..

[61] Thigpen JT, Blessing JA, Homesley H, Creasman WT, Sutton G (1989). Phase II trial of cisplatin as first-line chemotherapy in patients with advanced or recurrent endometrial carcinoma: A Gynecologic Oncology Group Study. Gynecol. Oncol..

[62] Covens AL (2011). Phase II study of fulvestrant in recurrent/metastatic endometrial carcinoma: A Gynecologic Oncology Group study. Gynecol. Oncol..

[63] Fiorica JV (2004). Phase II trial of alternating courses of megestrol acetate and tamoxifen in advanced endometrial carcinoma: A Gynecologic Oncology Group study. Gynecol. Oncol..

[64] Makker V (2019). Breaking new ground in the treatment of advanced endometrial cancer. Oncology (Williston Park).

[65] Hosny A, Parmar C, Quackenbush J, Schwartz LH, Aerts H (2018). Artificial intelligence in radiology. Nat. Rev. Cancer.

[66] Jorgensen JT (2016). Companion and complementary diagnostics: Clinical and regulatory perspectives. Trends Cancer.

