# Supplementary material for: Machine learning-based prediction of microsatellite instability and high tumor mutation burden from contrast-enhanced computed tomography in endometrial cancers
Source: Sci Rep. 2020 Oct 20;10:17769. doi: 10.1038/s41598-020-72475-9 (PMC7575573; doi:10.1038/s41598-020-72475-9)
Supplement: Supplementary file 1 — Supplementary Information. [file 41598_2020_72475_MOESM1_ESM.docx]

**Machine learning-based prediction of microsatellite instability and high tumor mutation burden from contrast-enhanced computed tomography in endometrial cancers**

Harini Veeraraghavan†^1^, Claire F. Friedman†^2,6^, Deborah F. DeLair^3a^, Josip Ninčević^,4b^, Yuki Himoto^4c^, Silvio G. Bruni^4d^, Giovanni Cappello^4e^, Iva Petkovska^4^, Stephanie Nougaret^4f,g^, Ines Nikolovski^4^, Ahmet Zehir^3^, Nadeem R. Abu-Rustum^5^, Carol Aghajanian^2,6^, Dmitriy Zamarin^2,6^, Karen A. Cadoo^2,6^, Luis A. Diaz Jr^2,6^, Mario M. Leitao Jr^5^, Vicky Makker^2,6^, Robert A. Soslow^3^, Jennifer J. Mueller^5^, Britta Weigelt‡^3^, Yulia Lakhman‡*^4^

^1^Departments of Medical Physics, ^2^Department of Medicine, ^3^Department of Pathology, ^4^Department of Radiology, ^5^Gynecology Service, Department of Surgery, Memorial Sloan Kettering Cancer Center, New York, NY, USA; ^6^Department of Medicine, Weill Cornell Medical College, New York, NY, USA; †*Equal contribution;* ‡*Joint direction of work; *Corresponding author.*

**Authors’ Current Affiliations:** ^a^Department of Pathology, NYU Langone Medical Center, New York, NY, USA; ^b^Department of Radiology, Sisters of Charity Hospital, Zagreb, Croatia, ^c^Department of Diagnostic Radiology, Japanese Red Cross Wakayama Medical Center, Wakayama, Japan; ^d^Department of Radiology, Trillium Health Partners, Mississauga, ON, Canada; ^e^Department of Radiology, Candiolo Cancer Institute, FPO-IRCCS, Candiolo, Turin, Italy; ^f^Department of Radiology, Institute of Cancer Research of Montpellier (IRCM), INSERM U1194, Montpellier, France; ^g^Department of Radiology, Montpellier Cancer Institute, University of Montpellier, Montpellier, France

**CT Imaging Acquisition**

Ninety-six (64%) of 150 CE-CTs were obtained at our institution; 54 (36%) were acquired at outside centers and digitized into the picture archiving and communication system (PACS, Centricity, GE Medical Systems). The CT scanner manufacturers were as follows: 1) GE Medical Systems (N = 115; 76.6%), 2) Siemens Healthineers (N = 28; 18.7%), 3) Toshiba Medical Systems Corporation (N = 4; 2.7%), and 4) Phillips Healthcare (N = 3; 2.0%). Most CE-CTs (N = 120; 80%) were acquired with a peak kilovoltage (kVp) of 120 (mean: 120 kVp, range:100–140) and reconstructed with the standard convolutional kernel using 5 mm slice thickness (mean: 4.73 mm, range: 2.5–5 mm).

**Details of Radiomic Features**

A total of 200 features (100 features from within the tumor VOI and 100 features from within the peritumoral-rim VOI) were computed.^1-8^ These were as follows:

1. *Moments of intensity-volume histogram (4 features)*

These features are computed by discretizing the intensities within the VOI into a histogram and included mean, standard deviation, kurtosis, and skewness. We used a bin size of 32 for feature computation.

1. *Gray level co-occurrence matrix, GLCM (5 Haralick features × 2)*

These features include energy, entropy, correlation, contrast, and homogeneity. These features capture the local intensity variation between neighborhood of voxels. We used a bin size of 32, offset distance of 1 voxel and one offset direction.

1. *Gray level run length matrix, GLRLM (13 features × 2)*

These features summarize the statistical relationship of voxels in the binary mask enclosing the VOI. These features consist of short run emphasis (SRE), long run emphasis (LRE), gray level non-uniformity (GLN), run length non-uniformity (RLN), run percentage (RP), low gray run length emphasis (LGRLE), high gray run length emphasis (HGRLE), short run low gray level emphasis (SRLGLE), short run high gray level emphasis (SRHGLE), long run low gray level emphasis (LRLGLE), long run high gray level emphasis (LRHGLE), gray level variance (GLV), and run length variance (RLV).

1. *Gray level size zone matrix, GLSZM (13 features × 2)*

These features summarize the intensity distribution within the binary mask enclosing the VOI by the distribution of clustered groups of iso-intense voxels. These features are computed from a size zone matrix computed using number of gray levels (N = 32), and zone size or number of considered neighboring voxels (N = 26 for 3D neighborhood). The computed features include: small area emphasis (SAE), large area emphasis (LAE), gray level non-uniformity (GLN), size zone-non uniformity (SZN), zone percentage (ZP), low gray level zone emphasis (LGLZE), high gray level zone emphasis (HGLZE), small area low gray level emphasis (SALGLE), small zone high gray level emphasis (SZHGLE), large area low gray level emphasis (LALGLE), large area high gray level emphasis (LAHGLE), gray level variance (GLV), and size zone variance (SZV).

1. *Neighborhood gray tone difference matrix, NGTDM (7 features × 2)*

These features quantify the variabilities in the gray level around a fixed neighborhood of voxels. These features are computed using a 3D neighborhood with a diameter of 3 *×* 3 *×* 3 pixels. The features include: coarseness, contrast, busyness, complexity, and strength.

In addition, two features called peak and valley features were computed.

1. *Neighborhood gray level dependence matrix, NGLDM (15 features × 2)*

These features quantify the textural variabilities by summarizing the images into a discretized intensity level-based matrix computed within a neighborhood at various intensity difference thresholds. The neighborhood size and intensity difference thresholds are automatically computed based on the VOI size and the intensities within the VOI. These features include: low dependence emphasis (LDE), high dependence emphasis (HDE), low grey level count emphasis (LGCE), high gray level count emphasis (HGCE), low dependence low gray level emphasis (LDLGE), low dependence high gray level emphasis (LDHGE), high dependence low gray level emphasis (HDLGE), high dependence high gray level emphasis (HDHGE), gray level non-uniformity (GLN), dependence count non-uniformity (DCN), gray level variance (GLV), dependence count percentage (DCP), dependence count entropy (DCEntropy), dependence count variance (DCV), and dependence count energy (DCEnergy).

1. Morphological shape features (9 features × 2)

These features summarize the statistics of the shape and include surface area, volume, filled volume, compactness, surface-to-volume ratio, sphericity, elongation, flatness, maximum three-dimensional diameter.

1. Sobel edge features (4 features × 2)

These features capture the mean, standard deviation, kurtosis, and skewness of the edges

1. Gabor filters at four orientations (0^o^, 45^o^, 90^o^, 135^o^) and two bandwidths ($\sqrt{2}$, 2$\sqrt{2}$) (32 features × 2)

**SUPPLMENTAL TABLES:**

| Supplemental Table 1. RADIOMIC FEATURES AND CLINICAL VARIABLES SELECTED BY THE RFE-RF CLASSFIER AS THE MOST RELEVANT  To identify and distinguish MMR-D from CN-low-like and CN-high-like subtypes of EC | | | | |
| --- | --- | --- | --- | --- |
| Features  (orientations, bandwidth) | MMR-D  Median (IQR) | CN-low-like +  CN-high-like  Median (IQR) | Adjusted  p-value | Gini  feature  Importance |
| Peritumoral-rim  Gabor_mean_ (135⁰, $2\sqrt{2}$) | 0.017  (−0.044, 0.073) | −0.036  (−0.106, 0.027) | 0.008 | 100 |
| Peritumoral-rim  Gabor_mean_ (0⁰, $2\sqrt{2}$) | 0.080  (−0.064, 0.224) | −0.077  (−0.121, 0.023) | < 0.001 | 48.9 |
| Peritumoral-rim  Gabor_mean_ (135⁰, $\sqrt{2}$) | 0.012  (−0.024, 0.067) | −0.034  (−0.079, 0.008) | < 0.001 | 32.3 |

***Abbreviations*:** MMR-D DNA mismatch repair-deficient; CN copy number; EC endometrial cancer; IQR interquartile range

***Note:*** P-values were adjusted for multiple comparisons.

| Supplemental Table 2. RADIOMIC FEATURES AND CLINICAL VARIABLES SELECTED BY THE RFE-RF CLASSFIER AS THE MOST RELEVANT  To distinguish TMB-H from TMB-L EC tumors | | | | | |
| --- | --- | --- | --- | --- | --- |
| Features  (orientations, bandwidth) | TMB high Median (IQR) | TMB low  Median (IQR) | Adjusted  p-value | Gini  feature  Importance | |
| Peritumoral-rim  Gabor_mean_ (135⁰, $2\sqrt{2}$) | 0.024  (−0.038, 0.42) | −0.040  (−0.106, 0.005) | 0.017 | | 100 |
| Peritumoral-rim  Gabor_mean_ (135⁰, $\sqrt{2}$) | 0.078  (−0.066, 0.220) | −0.079  (−0.120, 0.022) | 0.004 | | 82.9 |
| Peritumoral-rim Valley | 3.50  (−1.00, 16.80) | 2.00  (−5.30, 9.40) | 0.27 | | 67.6 |
| Peritumoral-rim Gabor_skew_(45⁰, $\sqrt{2}$) | 0.062  (0.012, 0.155) | 0.008  (−0.057, 0.080) | 0.039 | | 63.4 |
| Peritumoral-rim  Gabor_mean_ (45⁰, $\sqrt{2}$) | 0.093  (−0.066, 0.20) | −0.067  (−0.144, −0.005) | 0.001 | | 59.8 |
| DC Entropy | 8.40  (8.10, 8.60) | 8.20  (8.00, 8.30) | 0.044 | | 51.3 |
| Peritumoral-rim HGRLE | −6.2  (−40.3, 31.3) | 17.0  (−11.0, 60.0) | 0.024 | | 48.0 |
| Complexity | 958 (854, 1100) | 1060 (941, 1202) | 0.13 | | 47.4 |
| Peritumoral-rim Gabor_skew_(45⁰, 2$\sqrt{2}$) | 0.036  (−0.022, 0.14) | 0.002  (−0.070, 0.092) | 0.17 | | 47.2 |
| Peritumoral-rim HGCE | −5.6  (−40.3, 31.8) | 17.0  (−11.0, 61.0) | 0.024 | | 46.8 |
| Correlation | 0.64  (0.46, 0.72) | 0.52  (0.40, 0.62) | 0.024 | | 44.3 |
| Gabor_skew_(0⁰, 2$\sqrt{2}$) | −0.042  (−0.12, 0.044) | 0.001  (−0.078, 0.073) | 0.59 | | 44.3 |
| Age | 60 (53, 68) | 65 (60, 71) | 0.044 | | 44.0 |
| Peritumoral-rim SD | −1.76  (−4.25, −0.89) | −1.19  (−1.99, −0.55) | 0.05 | | 42.8 |
| Peritumoral-rim SRHGLE | −0.10  (−34.2, 32.2) | 19.4  (−9.4, 62.2) | 0.028 | | 42.0 |
| Peritumoral-rim HGLZE | −11.0  (−40.0, 21.0) | 11.0  (−15.0, 51.0) | 0.044 | | 38.9 |
| Peritumoral-rim Gabor_skew_(135⁰, 2$\sqrt{2}$) | 0.024  (−0.045, 0.133) | −0.039  (−0.136, 0.028) | 0.006 | | 32.8 |
| Peritumoral-rim Gabor_skew_(135⁰, $\sqrt{2}$) | 0.027  (−0.067, 0.178) | −0.039  (−0.15, 0.05) | 0.076 | | 32.0 |
| Peritumoral-rim SZHGLE | 5.60  (−10.0, 25.7) | 17.9  (−2.70, 41.6) | 0.13 | | 31.4 |
| Peritumoral-rim Skewness | 0.009  (−0.077, 0.130) | −0.051  (−0.168, 0.022) | 0.05 | | 29.7 |
| Peritumoral-rim  RLN | -56,369  (−103,066, −30,319) | −38,831  (−89,713, −23,919) | 0.59 | | 28.2 |

***Abbreviations:*** DC Entropy dependence count entropy; TMB tumor mutational burden; TMB-H TMB-high; TMB-L TMB low; EC endometrial cancer; IQR interquartile range; HGRLE high gray run length emphasis; HGCE high gray level count emphasis; SD standard deviation; SRHGLE short run high gray level emphasis; HGLZE high gray level zone emphasis; SZHGLE Small zone high gray level emphasis; RLN run length non-uniformity

**REFERENCES:**

1. Apte AP, Iyer A, Crispin-Ortuzar M, et al: Technical Note: Extension of CERR for computational radiomics: A comprehensive MATLAB platform for reproducible radiomics research. Med Phys, 2018

2. Haralick RM, Shanmuga.K, Dinstein I: Textural features for image classification. Ieee Transactions on Systems Man and Cybernetics SMC3:610-621, 1973

3. Galloway M: Texture analysis using gray level run lengths. Computer Vision Graphics and Image Processing 4:172–179, 1975

4. Thibault G, Fertil B, Navarro C, et al: Shape and Texture Indexes Application to Cell Nuclei Classification. International Journal of Pattern Recognition and Artificial Intelligence 27, 2013

5. Amadasun M, King R: Textural features corresponding to textural properties. IEEE Transactions on Systems, Man, and Cybernetics 19:1264-1274, 1989

6. Sun CJ, Wee WG: Neighboring Gray Level Dependence Matrix for Texture Classification. Computer Vision Graphics and Image Processing 23:341-352, 1983

7. Sobel I: An isotropic 3×3 gradient operator, Academic Press, 1990 pp. 376–379

8. Daugman JG: Uncertainty relation for resolution in space, spatial frequency, and orientation optimized by two-dimensional visual cortical filters. J Opt Soc Am A 2:1160-9, 1985
